# Supplementary material for: Stress-induced phase separation in plastics drives the release of amorphous polymer micropollutants into water
Source: Nat Commun. 2025 Apr 23;16:3814. doi: 10.1038/s41467-025-58898-w (PMC12018937; doi:10.1038/s41467-025-58898-w)
Supplement: Supplementary file 1 — Supplementary Information [file 41467_2025_58898_MOESM1_ESM.pdf]

Supplementary Information for

Stress-induced phase separation in plastics drives the release of amorphous polymer micro-pollutants into water

Dunzhu Li[1,2,3]*, Peijing Li[4], Yunhong Shi[3], Emmet D. Sheerin[2,5], Zihan Zhang[3], Luming Yang[3,2], Liwen Xiao[3,6]*, Christopher Hill[2,5], Conall Gordon[2,5], Manuel Ruether[5], Joshua Pepper[2,5], John E. Sader[7], Michael A. Morris[2,5], Jing Jing Wang[2]*, John J. Boland[2,5]*

[1]Jiyang College, Zhejiang A&F University; Zhuji, China.

[2]AMBER Research Centre and Centre for Research on Adaptive Nanostructures and Nanodevices (CRANN), Trinity College Dublin; Dublin, Ireland.

[3]Department of Civil, Structural and Environmental Engineering, Trinity College Dublin; Dublin, Ireland.

[4]School of Mathematics and Statistics, The University of Melbourne; Victoria, Australia.

[5]School of Chemistry, Trinity College Dublin; Dublin, Ireland.

[6]TrinityHaus, Trinity College Dublin; Dublin, Ireland.

[7]Graduate Aerospace Laboratories and Department of Applied Physics, California Institute of Technology; Pasadena, USA.

*Correspondence to: Dunzhu Li, lidu@tcd.ie; Liwen Xiao, liwen.xiao@tcd.ie; Jing Jing Wang, jjwang@tcd.ie; John J. Boland, jboland@tcd.ie

Table of Contents

Supplementary Notes 3
Supplementary Figures 1-16
Supplementary Tables 1-2
Supplementary references

Supplementary Note 1 Determination of materials' crystallinity

XRD analysis revealed that the crystallinity degree of the standard PP sheet was 49.7°. The Raman spectra distinctly showed the crystalline C-C backbone vibration at 1168 cm^{-1}. Additionally, two sharp peaks at 810 and 840 cm^{-1}, associated with helical chains within the crystals, were observed, aligning with previous reports[1,2]. Combined with the XRD results, it is evident that the standard PP sheet is semi-crystalline, containing both crystalline and amorphous regions.

Using the same system setting and HQI index (Method in the main text), the spectra of separated droplets (the surface droplets were transferred to an aluminium foil to avoid spectral interference from the bulk PP sheet), plastic sheets and products were obtained and compared with standard plastic spectra. The full range (250-3500 cm^{-1}, Suppl. Fig. 5) HQI of separated droplets compared to the parent semicrystalline PP and standard amorphous PP wax (Goodfellow) was 0.97 and 0.98, respectively. The high HQI values confirm the a-PP nature of the separated droplets. Notably, Raman spectra in Fig. 1c clearly showed that the droplets perfectly matched that of standard amorphous PP wax, with a hit quality index (HQI) of 0.93 at low wavenumber range (800-1500 cm^{-1}, Fig. 1c). Interestingly, there were clear differences between PP droplets and the parent PP cantilever beam at low wavenumber (800-1500 cm^{-1}), with a substantial difference at 810 and 840 cm^{-1} associated with the crystallinity degree of PP. Additionally, PP droplets have no peak at 1168 cm^{-1} associated with the crystalline C-C backbone vibration [1,2] (Fig. 1c). Those differences gave rise to a poor match between PP droplets and the parent PP cantilever, with an HQI of only 0.75. Similar difference was observed in FTIR spectra (Suppl. Fig. 6). FTIR is a complementary tool that can further confirm the amorphous nature of the droplets. Different from Raman spectroscopy, FTIR is more sensitive at the peaks of 997 and 973 cm^{-1}, which are primarily associated with the rocking of crystalline C-C backbone and the stretching of CH_3, respectively [3-5]. The crystallinity degree of PP is proportional to the ratio between the peak intensity of 997 and 973 cm^{-1} [4,5]. Clearly, the intensity of 997 cm^{-1} is negligible, which closely matched the spectrum of standard amorphous PP. Evidently, most of these droplets are a-PP that has been squeezed out from the bulk PP sheet.

Supplementary Note 2 Determination of materials' molecular weight

The average molecular weight (MW) associated with the minor peak (Fig. 1d), which accounts for 21% of the total mass of the droplets, is approximately 510 g mol^{-1}, corresponding to a degree of polymerization (n) of 12. Currently, information on the size cutoff between oligomers and polymers remains limited, although various regulations and research studies have used size cutoffs ranging from several to 40 repeat units[6-8]. Although migration of low molecular weight substances was not the predominant phenomenon observed in this study, the co-migration of oligomers and additives with polymers could be accelerated under harsh conditions, such as exposure to microwave heating or hot oil soaking. This indicates the necessity for further research to elucidate synergistic impacts of exposed conditions and surface stress on the migration behaviors of each component.

Supplementary Note 3 Investigation of standard polyethylene sheet

In addition to PP plastic sheets, we investigated stress-driven amorphous phase separation on the surfaces of standard semicrystalline polyethylene (PE) sheets (Suppl. Fig. 14). By configuring a PE sheet into a cantilever setup (Suppl. Table 1), we observed that only the upper compressive side of the cantilever, approximately 3 mm from the clamped end, developed a significant number of droplets. These droplets typically ranged from 10-40 µm in lateral size and 0.3-2 µm in height after being exposed to a 95 °C oven (Suppl. Fig. 14e-g). In contrast, no observable changes occurred on the tensile surface or the compressive side near the free end, consistent with observations from PP sheets. Raman spectroscopy analysis indicated the absence of the peak at 1416 cm^{-1}, typically associated with methylene bending[9]. The degree of crystallinity in PE is also proportionally related to the intensity of this 1416 cm^{-1} peak (Suppl. Fig. 14c)[9]. The absence of this specific band confirmed that the migrated droplets from the PE sheet were also amorphous. Following the same experimental protocol as with the PP sheet, the normalized volumetric flow ($\hat{v}$) of amorphous PE (a-PE) droplets was obtained as 26.6 $nm^3(nm^2 \cdot h)^{-1}$ or nm h^{-1}, which is higher than that observed for PP under the same compressive stress level (around -5 MPa, Fig. 2c), showing that the specific nature of polymer material influences the migration speed of amorphous polymer. Additionally, PE sheets formed into a cylindrical shape were also immersed in 95 °C DI water for 4 hours. Post-exposure inspection revealed a high quantity of circular shapes formed by air bubbles on the surface of the PE sheet (Suppl. Fig. 14c). Residues of these partial circles were also observed, indicating dynamic processes of air bubble expansion and coalescence that substantially deformed these separated droplets. No a-PE droplets or circles were observed on the outer tensile surface of the cylindrical sheets.

Supplementary Figures

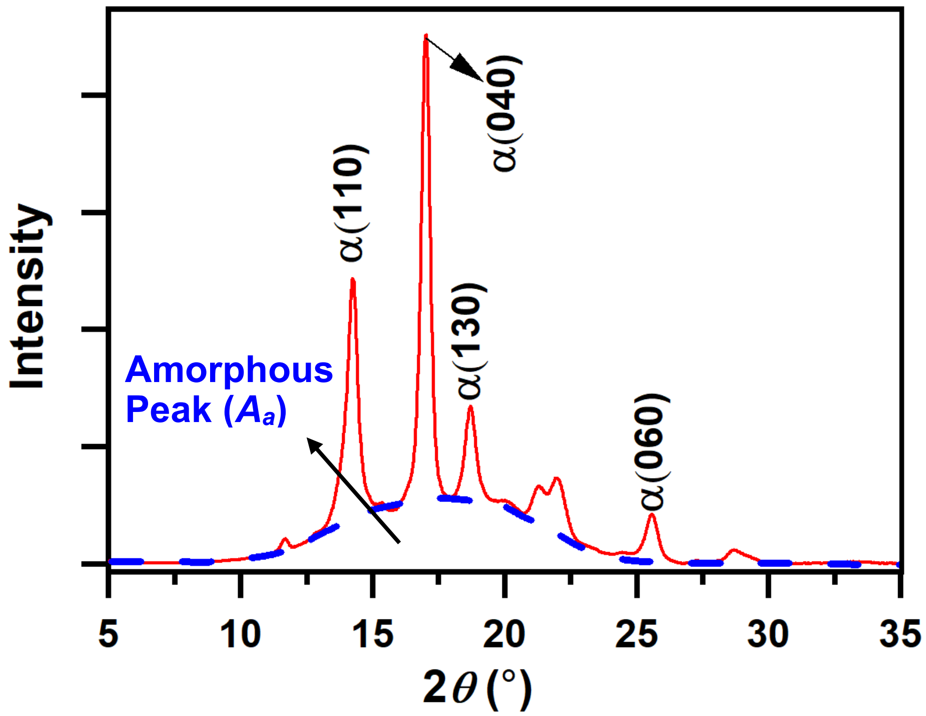

Supplementary Figure 1 XRD pattern of the standard semicrystalline PP sheet.

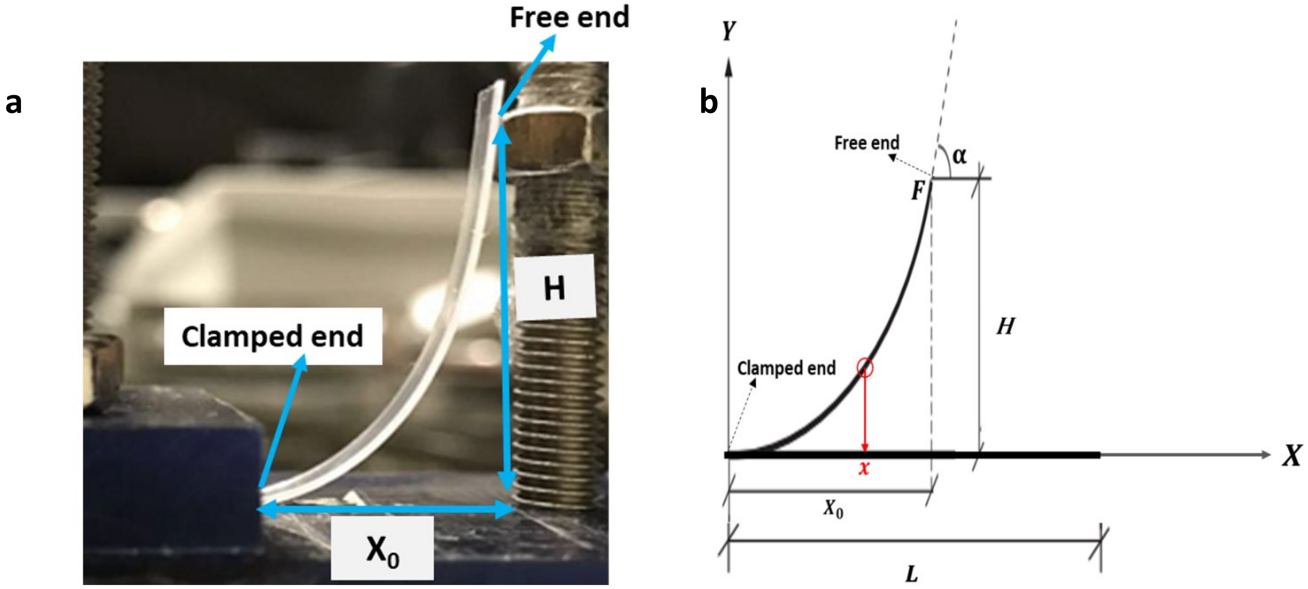

Supplementary Figure 2 Experimental Setup and Schematic Diagram of the cantilever. (a) The typical setting of cantilever using standard PP and PE sheet; (b) The diagram of cantilever. For the cantilever PP-1 setting (Suppl. Table 1) used in most experiments, the deflection height *H*, horizontal length X_0 and PP sheet length between free end and clamped end *L* were 20, 16 and 28 mm, respectively. Cantilever experiments that involved other dimensions are detailed in Suppl. Table 1.

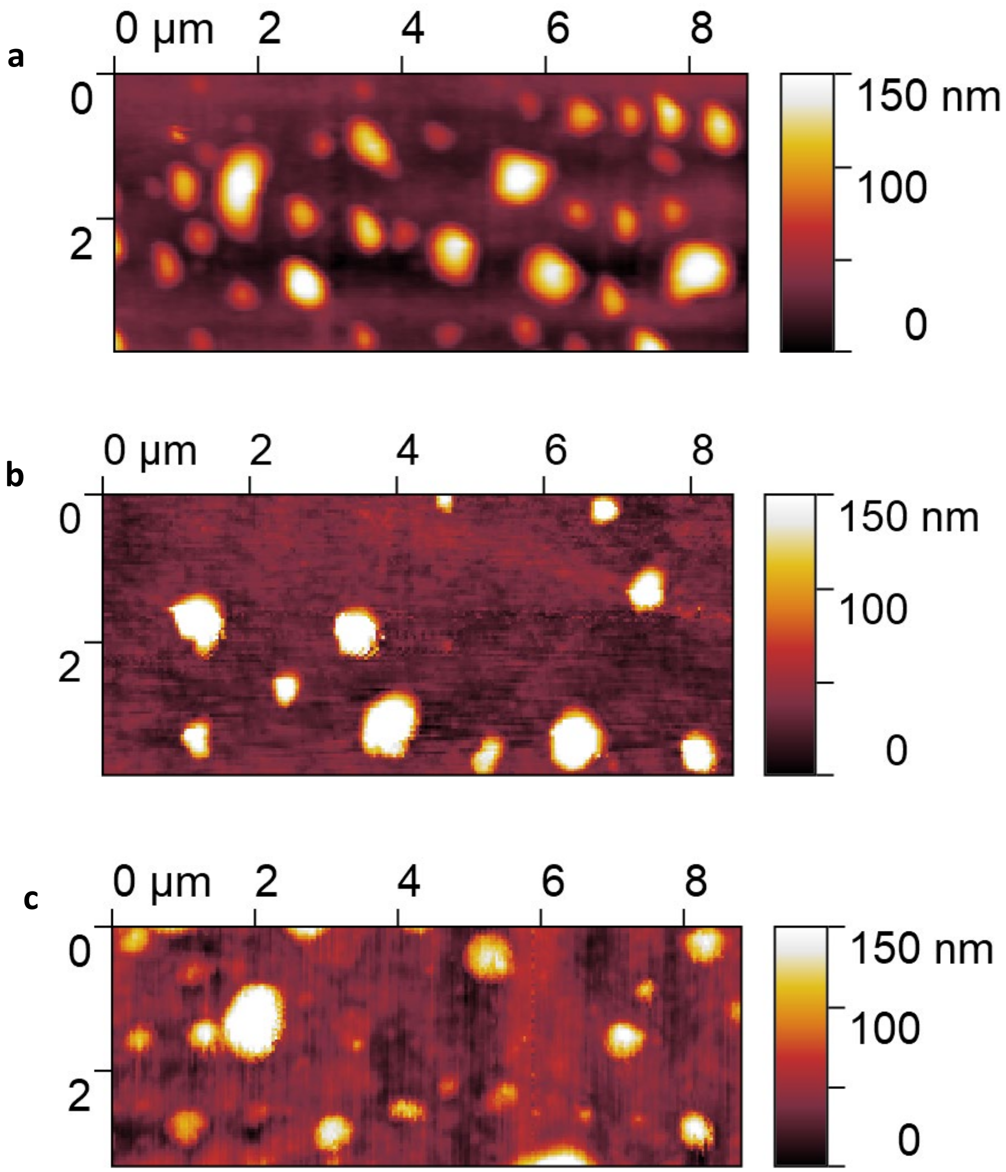

Supplementary Figure 3 Surface stress drives amorphous polypropylene separation from semicrystalline polypropylene sheets at different temperatures and time durations. (a) AFM height image of compressive side 5 mm from the clamped end after 7 h of 85 °C oven heat; (b) AFM height image of compressive side 0.5 mm from the clamped end after 120 h of 60 °C oven heat. PP sheets in Figs. a and b were compressed using cantilever PP-1 in Suppl. Table 1; (c) AFM height image of compressive side 0.1 mm from clamped end after 40 days of 35 °C oven heat, compressed using a shorten cantilever PP-2 in Suppl. Table 1.

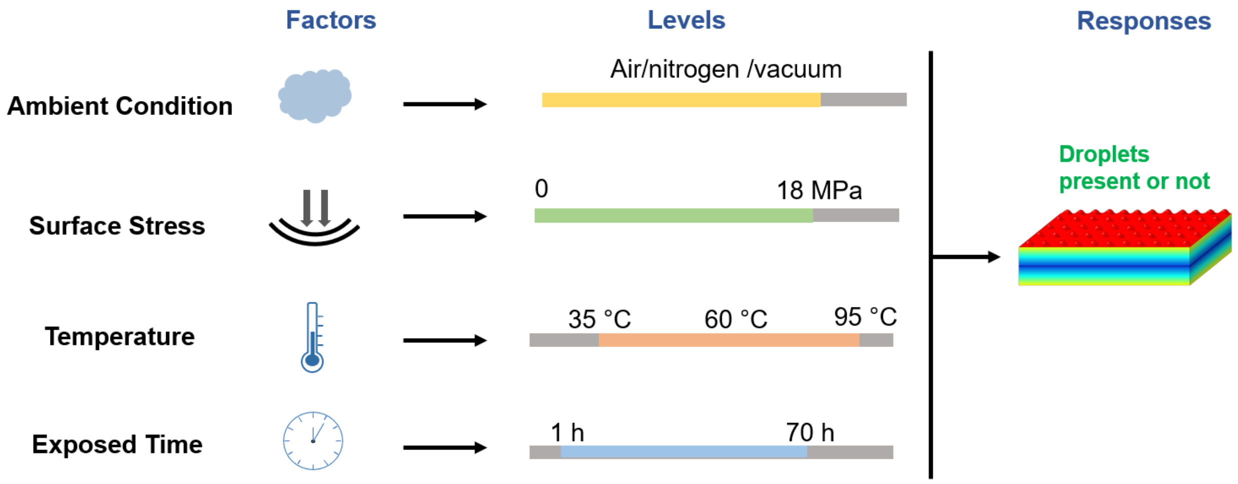

Supplementary Figure 4 The design of experiments using a full factorial methodology with four factors.

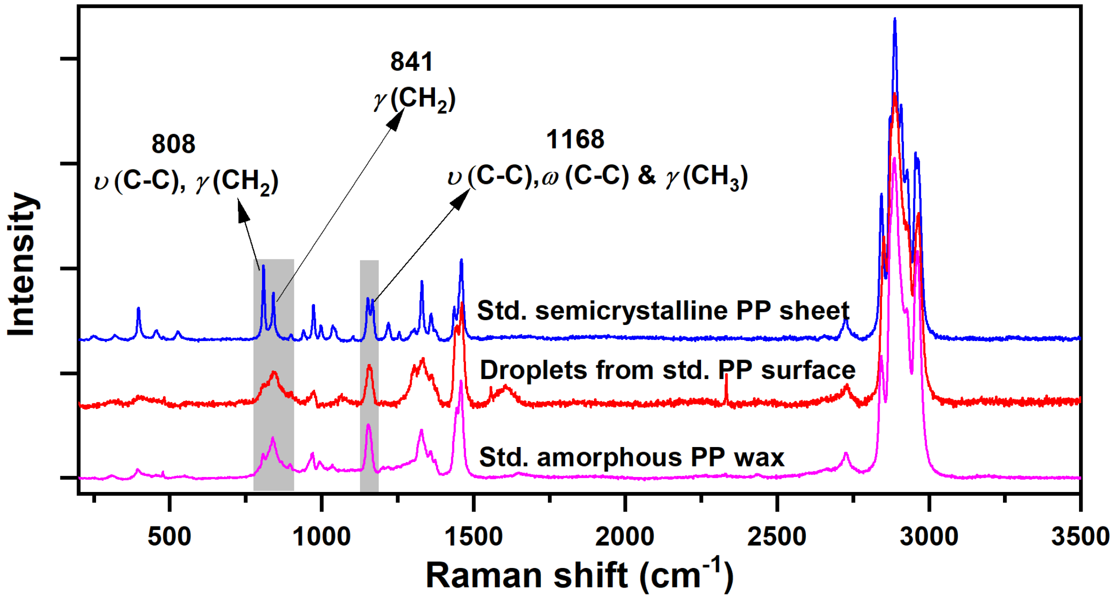

Supplementary Figure 5 Full Raman spectra of standard (std.) bulk PP sheet, surface droplets from bulk PP sheet and standard amorphous PP wax, respectively. During Raman detection, the bulk standard samples of PP sheet and PP wax were detected directly. To avoid interference from the parent PP sheet the sticky gel-like droplets were transferred to an aluminium foil and their Raman spectrum recorded.

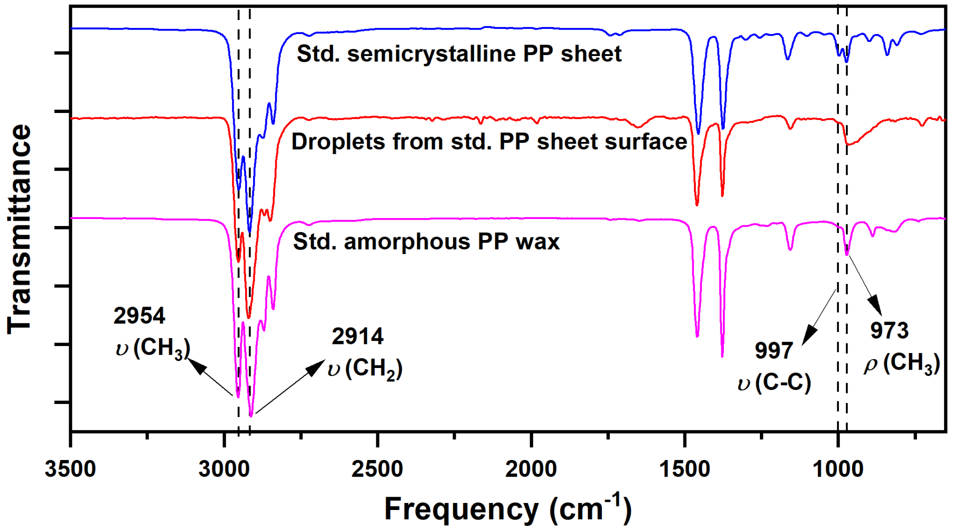

Supplementary Figure 6 FTIR spectra of standard (std.) bulk PP sheet, surface droplets from bulk PP sheet and standard amorphous PP wax, respectively. Similar to Raman detection, the sticky gel-like droplets were transferred to an aluminium foil to facilitate detection.

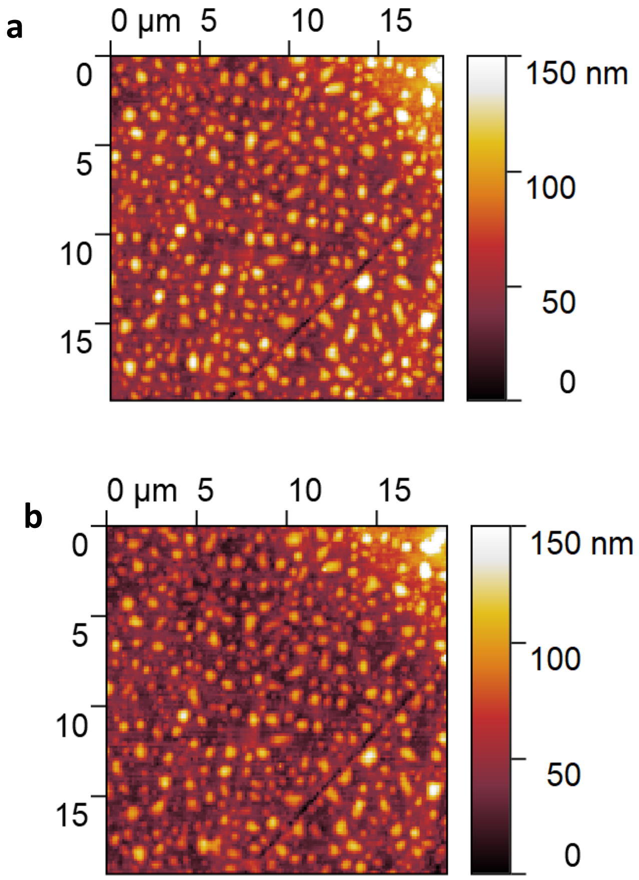

Supplementary Figure 7 In-situ test of the influence of ethanol exposure on droplets. (a) Droplets formed on standard PP sheet surface after 2 h of 95 °C oven heat in the shape of cantilever with setting 1 in Suppl. Table 1. (b) The same location after 5 h of ethanol soaking. Slight changes were observed, which confirmed that these droplets contain very low levels of organic additives that are ethanol soluble.

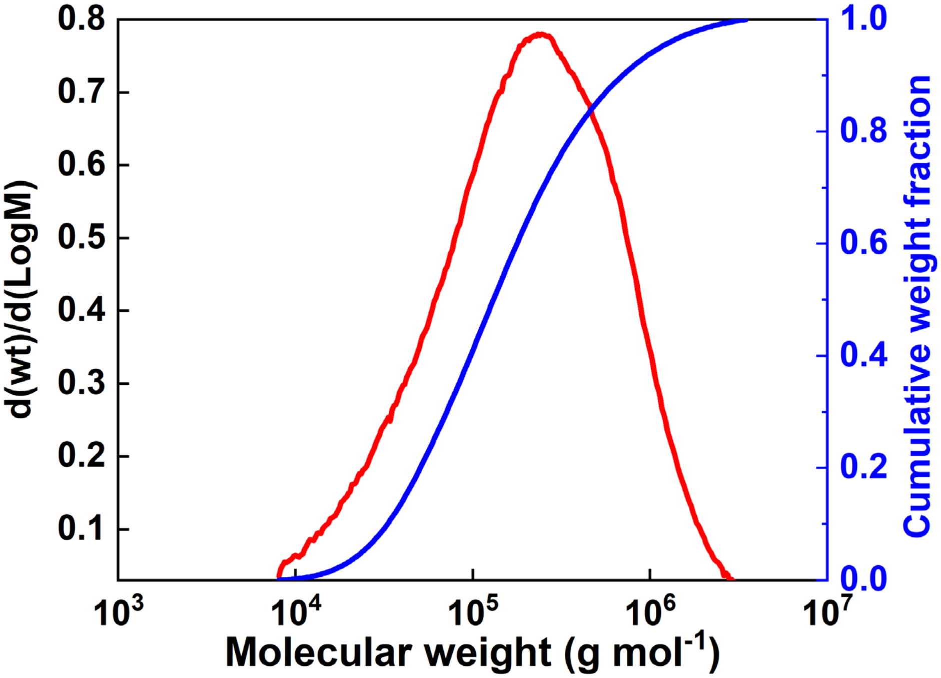

Supplementary Figure 8 The molecular weight (MW) and molecular weight distribution (MWD) of the standard semicrystalline PP sheet. Gel Permeation Chromatography (GPC, Agilent 1260 Infinity II GPC/SEC) was utilized to perform the test. During the test, the sample was dissolved using 1,2,4-Trichlorobenzene[10] (TCB, stabilized with butylated hydroxytoluene-BHT) while the column and detector temperatures were maintained at 150 °C. Other parameters were maintained the same as detailed in the main text. The number average molecular weight and the weight average molecular weight was 97,000 and 354,000 g mol^{-1}, respectively. The polydispersity index (PDI) was 3.6.

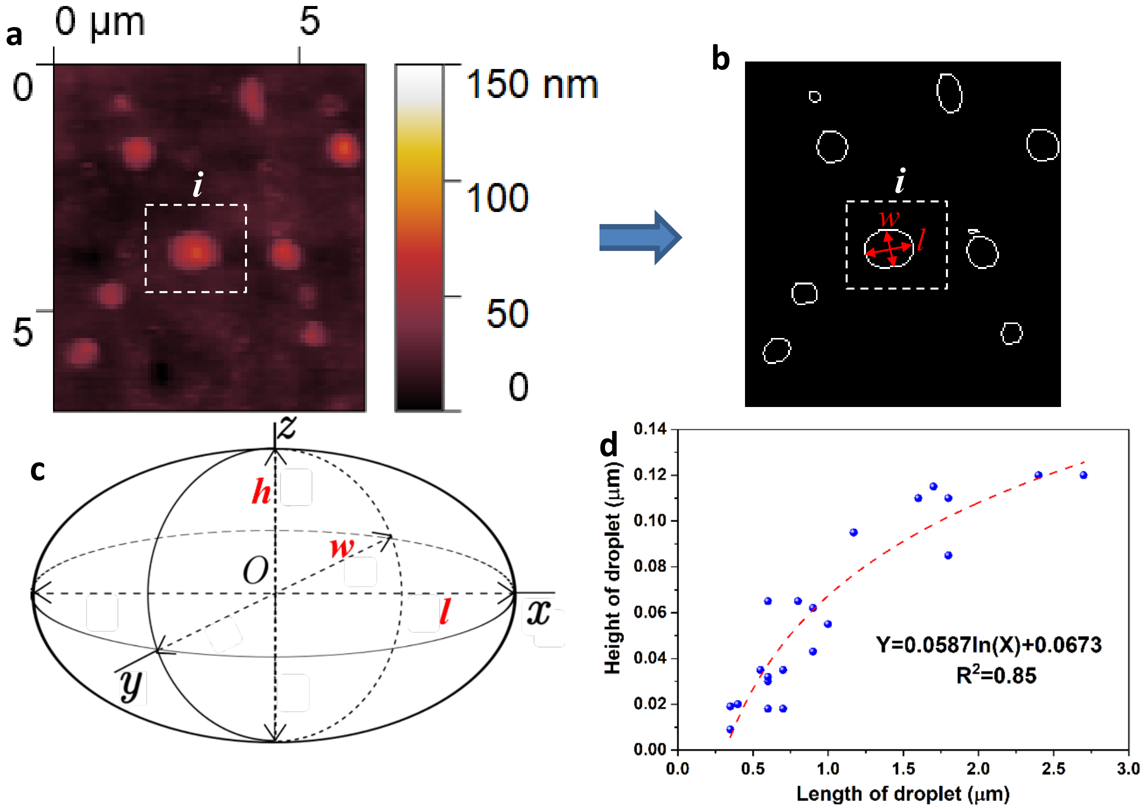

Supplementary Figure 9 Determination the volume of phase separated a-PP droplets. (a) A typical AFM image of separated droplets on PP cantilever surface after 4 h of 95 °C oven heat; (b) A typical analysis of image A using software of ImageJ to obtain the length (*l*) and width (*w*) of a separated droplet *i* in white box; (c) The ellipsoid shape used in the analysis of each droplet; (d) The correlation between the length (*l*) and height (*h*) of separated droplets, data obtained from the cross-section analysis of droplets' AFM images. In the fitting equation, Y represents height (*h*) and X represents lateral length (*l*). The high R^2 value indicates the reliability of the fit. The coefficients 0.0587 and 0.0673 are expressed in micrometers (µm).

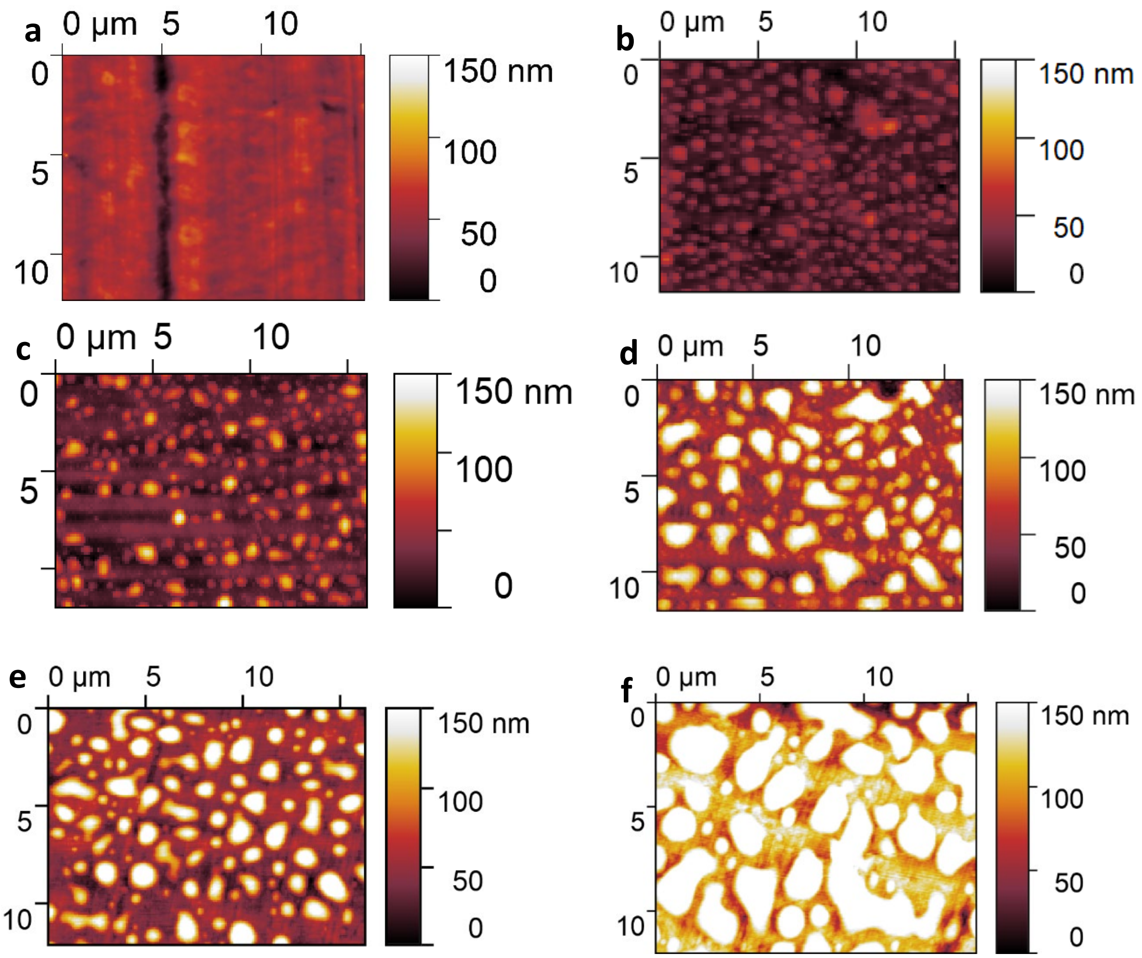

Supplementary Figure 10 Time dependence of the surface stress driven a-PP separation on the surface of a semicrystalline polypropylene sheet. AFM height image of compressive side (a) 12 mm and (b) 27.9 mm from the free end after 0.5 h of 95 °C oven heat, respectively; (c) and (d) are AFM height image of compressive side 12 mm and 27.9 mm from the free end after 4 h of 95 °C oven heat, respectively; (e) and (f) are AFM height image of compressive side 12 mm and 27.9 mm from the free end after 12 h of 95 °C oven heat, respectively.

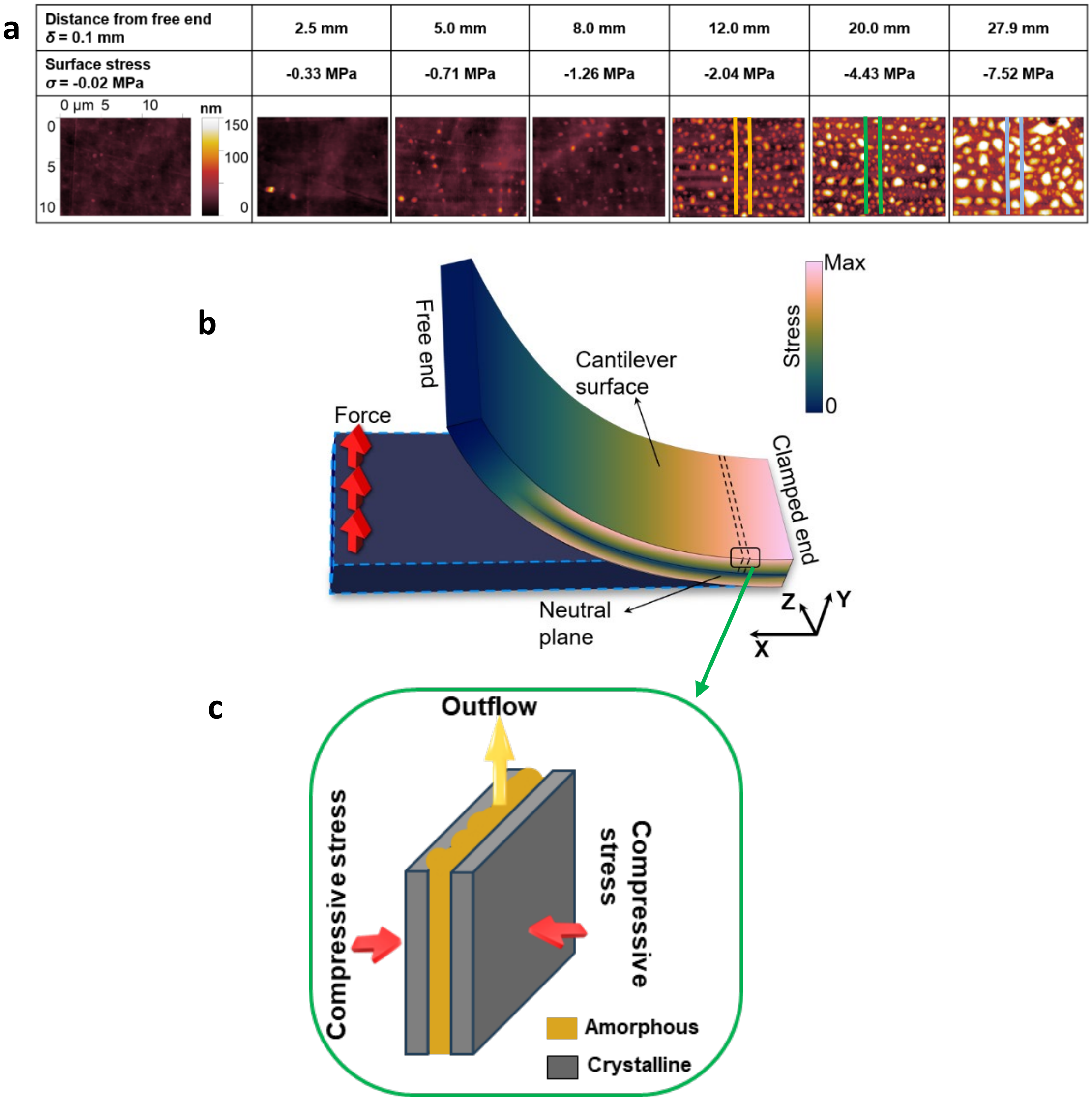

Supplementary Figure 11 Schematic diagram of the underlying stress-driven a-PP release mechanism in the cantilever experiment. (a) AFM images of the cantilever surface topography. Orange, green and blue solid lines represent some selected regions of the cantilever that are analyzed below; (b) Schematic showing a cross section of the cantilever from the neutral yz plane to the surface. Compressive stress (red arrows) increases from zero at the neutral plane to a maximum at the cantilever surface. Regions near the cantilever surface (green) bounded by faces in the xz plane are under compressive stress due to bending that in turn results to a-PP outward flow towards the surface; (c) Zoomed-in view of the selected region (circled in green) close to the cantilever surface. Faces in black represent the crystalline phase and yellow region represents the amorphous phase. Red arrow indicates the compressive stress acting on the crystalline surface. In the vicinity of the cantilever surface the compressive stress is approximately uniform across a short length scale of crystalline blocks with the semicrystalline plastic.

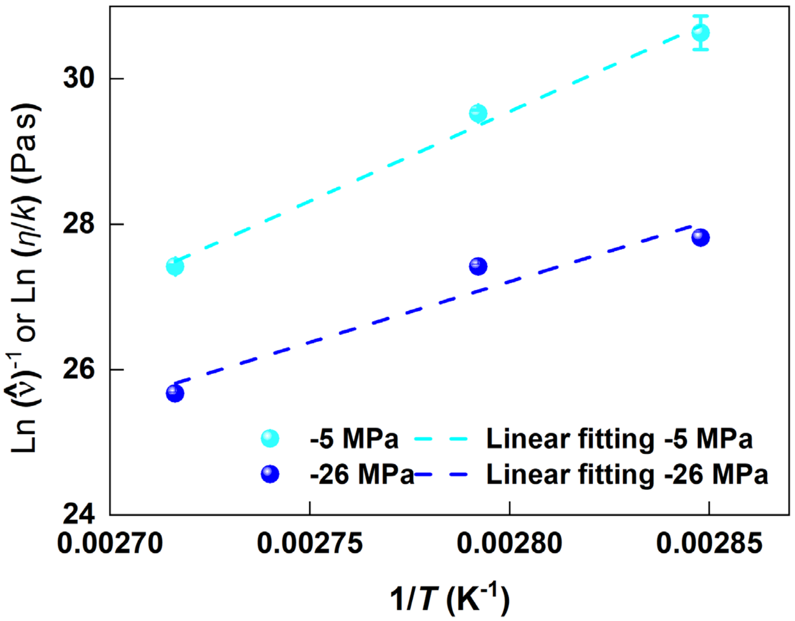

Supplementary Figure 12 Arrhenius plots of Eq. 2 at compressive surface stresses of -5 and -26 MPa respectively. Data extracted from 3 temperatures (78, 85 and 95 °C) in Fig. 2c. The slope of linear fitting at each stress can be used to calculate flow activation energy.

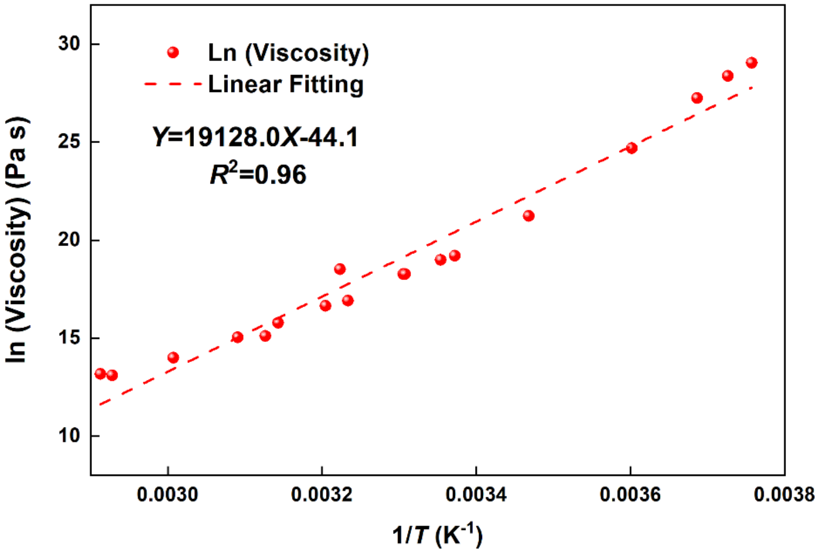

Supplementary Figure 13 The correlation between 1/T and Ln (Viscosity) of amorphous PP in previous publication. The fitting coefficients (19128.0 and 44.1) are dimensionless. Data were extracted from the publication of [11,12]. The flow activation energy (E_f) can be obtained multiply the slope by gas constant (R, 0.00831434 kJ mol^{-1} K^{-1}), which is 159.0 kJ mol^{-1}.

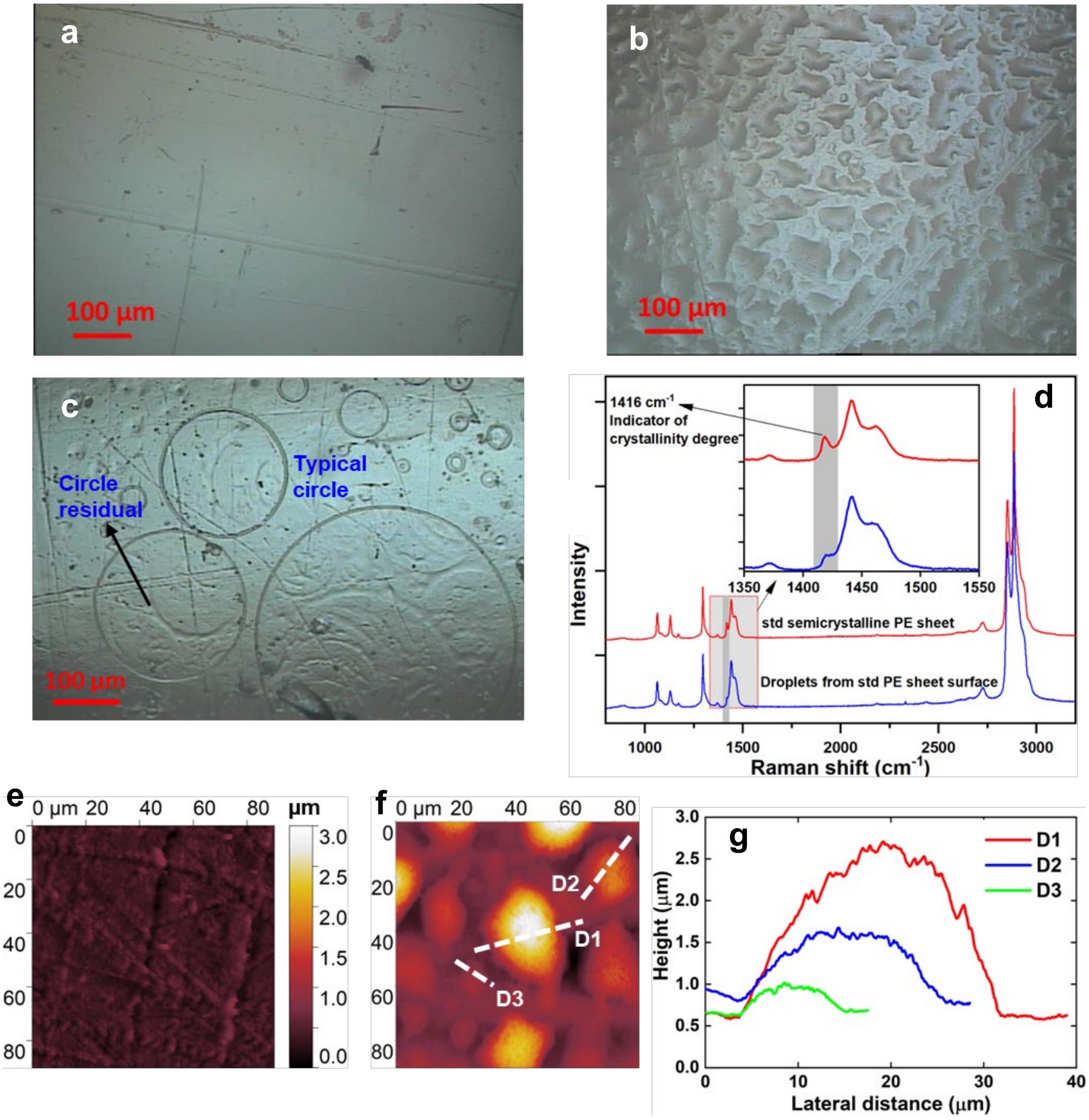

Supplementary Figure 14 Analysis of surface stress driven a-PE phase separation and rearrangement in water environment. a, Optical image of virgin surface of standard PE sheet (low density). b, Optical image of PE compressive surface that close to clamped end after 95 °C oven heat. c, Optical image of inner compressed surface of PE sheet after 4h of 95 °C hot water exposure. d, Raman spectra of droplets from the surface of the stressed PE sheet, compared to the spectra from standard bulk PE sheet. e-f, AFM images of PE surface before and after 95 °C oven heat, both images have the same scale bars. g, The cross-section profiles of typical surface droplets in Suppl. Fig. 14f. It was obtained under around -5 MPa of compressive stress at 95 °C, which is comparable to that of Fig 2a at a stress of -4.4 MPa.

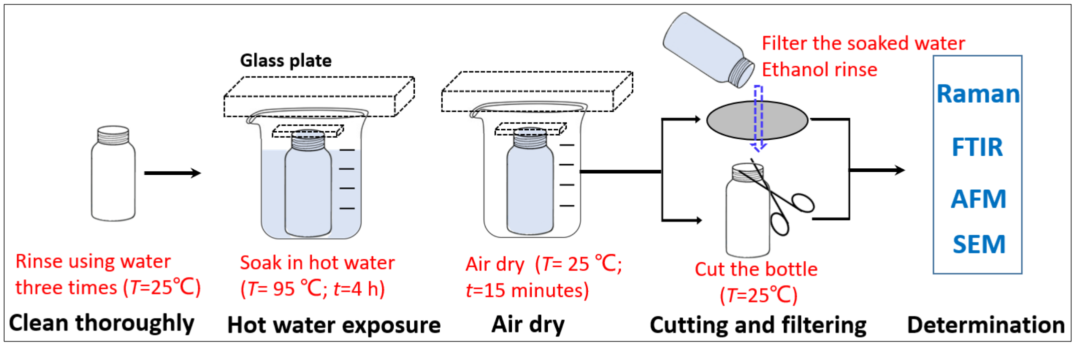

Supplementary Figure 15 Protocol to expose PP bottle to 95 °C water.

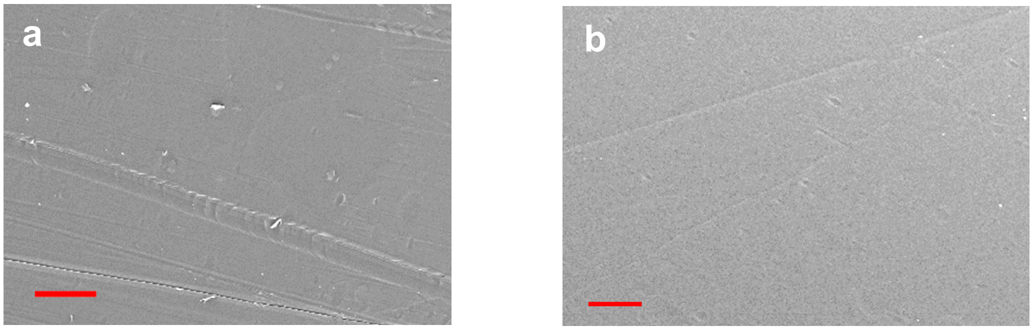

Supplementary Figure 16 (a) and (b), SEM images of the inner surface of bottle’s body before and after exposure to 95 °C water, respectively. The red scale bar in both figs is 5 µm.

Supplementary Tables

Supplementary Table 1 The summary of cantilever settings and test conditions

Sample type	Cantilever length, *L*	Cantilever horizontal length, *X*_0	Cantilever height (*H*)	Test temperature/ time
	mm	mm	mm	°C/h
Standard PP sheet-setting PP-1	28	16	20	95 °C/1, 4, 12 and 70 h; 85 °C/7 h and 70 h; 78 °C/12 and 70 h; 70 °C/19 and 70 h; 60 °C/70 h and 120 h
Standard PP sheet-setting PP-2	6	3.2	5	95 °C/0.33 h; 85 °C/1 h; 78 °C/2 h; 70 °C/3 h; 60 °C/70 h; 35 °C/40 days
Standard PE sheet-setting PE-1	6	3.2	5	95 °C/10 and 24 h

Cantilever length *L* is the physical distance between free end and clamped end of the plastic sheet.

Supplementary Table 2 The summary of statistical design of experiments (DoE)

FACTORS	DEGREE OF FREEDOM	*F*-VALUE	*P*-VALUE
**AMBIENT CONDITION**	2	0	1
**SURFACE STRESS**	1	58	0
**TEMPERATURE**	2	14.5	0
**EXPOSED TIME**	1	0	1

Supplementary references:

1 Nielsen, A. S., Batchelder, D. & Pyrz, R. Estimation of crystallinity of isotactic polypropylene using Raman spectroscopy. *Polymer* **43**, 2671-2676 (2002).

2 Gopanna, A., Mandapati, R. N., Thomas, S. P., Rajan, K. & Chavali, M. Fourier transform infrared spectroscopy (FTIR), Raman spectroscopy and wide-angle X-ray scattering (WAXS) of polypropylene (PP)/cyclic olefin copolymer (COC) blends for qualitative and quantitative analysis. *Polym. Bull.* **76**, 4259-4274 (2019).

3 McDonald, M. & Ward, I. The assignment of the infra-red absorption bands and the measurement of tacticity in polypropylene. *Polymer* **2**, 341-355 (1961).

4 Longo, C., Savaris, M., Zeni, M., Brandalise, R. N. & Grisa, A. M. C. Degradation study of polypropylene (PP) and bioriented polypropylene (BOPP) in the environment. *Mater. Res.* **14**, 442-448 (2011).

5 Paukkeri, R. & Lehtinen, A. Thermal behaviour of polypropylene fractions: 1. Influence of tacticity and molecular weight on crystallization and melting behaviour. *Polymer* **34**, 4075-4082 (1993).

6 European Chemicals Agency (ECHA). How to decide whether a substance is a polymer or not and how to proceed with the relevant registration. (Accessed by 2017.12).

7 Yang, T., Xu, Y., Liu, G. & Nowack, B. Oligomers are a major fraction of the released submicrometre particles released during washing of polyester textiles. *Nat. Water* **2**, 151-160 (2024).

8 International Organization for Standardization.. Biological evaluation of medical devices—. *Biol. Eval. Med. Devices* **1**, 10993 (2003).

9 Strobl, G. & Hagedorn, W. Raman spectroscopic method for determining the crystallinity of polyethylene. *Journal of Polymer Science: Polymer Physics Edition* **16**, 1181-1193 (1978).

10 Liu, Y., Bo, S., Zhu, Y. & Zhang, W. Determination of molecular weight and molecular sizes of polymers by high temperature gel permeation chromatography with a static and dynamic laser light scattering detector. *Polymer* **44**, 7209-7220 (2003).

11 Oßmann, B. E. *et al.* Small-sized microplastics and pigmented particles in bottled mineral water. *Water Res.* **141**, 307-316 (2018).

12 Imhof, H. K. *et al.* Pigments and plastic in limnetic ecosystems: A qualitative and quantitative study on microparticles of different size classes. *Water Res.* **98**, 64-74 (2016).
